# Metis: a python-based user interface to collect expert feedback for generative chemistry models

**DOI:** 10.1186/s13321-024-00892-3

**Published:** 2024-08-14

**Authors:** Janosch Menke, Yasmine Nahal, Esben Jannik Bjerrum, Mikhail Kabeshov, Samuel Kaski, Ola Engkvist

**Affiliations:** 1 Department of Computer Science and Engineering, Chalmers University of Technology, Gothenburg, 41296 Sweden; 2 Department of Computer Science, Aalto University, Espoo, 02150 Finland; 3 Cheminformania Consulting, Mölndal, 43138 Sweden; 4 Molecular AI, Discovery Sciences AstraZeneca R &D, Mölndal, 43183 Sweden; 5 Department of Computer Science, University of Manchester, Manchester, M13 9PL UK

**Keywords:** De novo drug design, Preference learning, Human-in-the-loop, Machine learning, User interface

## Abstract

One challenge that current de novo drug design models face is a disparity between the user’s expectations and the actual output of the model in practical applications. Tailoring models to better align with chemists’ implicit knowledge, expectation and preferences is key to overcoming this obstacle effectively. While interest in preference-based and human-in-the-loop machine learning in chemistry is continuously increasing, no tool currently exists that enables the collection of standardized and chemistry-specific feedback. Metis is a Python-based open-source graphical user interface (GUI), designed to solve this and enable the collection of chemists’ detailed feedback on molecular structures. The GUI enables chemists to explore and evaluate molecules, offering a user-friendly interface for annotating preferences and specifying desired or undesired structural features. By providing chemists the opportunity to give detailed feedback, allows researchers to capture more efficiently the chemist’s implicit knowledge and preferences. This knowledge is crucial to align the chemist’s idea with the de novo design agents. The GUI aims to enhance this collaboration between the human and the “machine” by providing an intuitive platform where chemists can interactively provide feedback on molecular structures, aiding in preference learning and refining de novo design strategies. Metis integrates with the existing de novo framework REINVENT, creating a closed-loop system where human expertise can continuously inform and refine the generative models.

**Scientific contribution**

We introduce a novel Graphical User Interface, that allows chemists/researchers to give detailed feedback on substructures and properties of small molecules. This tool can be used to learn the preferences of chemists in order to align de novo drug design models with the chemist’s ideas. The GUI can be customized to fit different needs and projects and enables direct integration into de novo REINVENT runs. We believe that Metis can facilitate the discussion and development of novel ways to integrate human feedback that goes beyond binary decisions of liking or disliking a molecule.

## Introduction

De novo drug design, a process of creating novel molecular structures with desired biological properties, stands as a cornerstone in the automation of the drug discovery process [1]. It often makes use of reinforcement learning to iteratively optimize molecules to achieve predefined objectives, such as efficacy and safety profiles [2, 3]. Reinforcement learning (RL) is a paradigm in machine learning that involves training agents to make sequences of decisions in an environment to maximize a cumulative reward. RL has demonstrated remarkable success across a wide range of applications outside of chemistry, enabling agents to learn highly complex behaviors [4–6]. A key to the successful training of an RL agent lies in the design of a well-defined reward function, which serves as a guide for the agent to achieve desirable behaviors. Without a carefully crafted reward function, the agents might not converge to desired behaviors. Additionally, there is a risk of reward exploitation and hacking, where the agent may find unintended shortcuts to maximize its rewards, leading to an agent maximizing the reward in an undesirable fashion [7, 8].

Creating a well-specified reward function is not only difficult as it requires a deep understanding of the task at hand, but the translation of that domain expertise into a parametric function used to compute the reward can pose a challenge. In many cases, domain experts struggle with the translation part, as they have a good idea of what an acceptable solution looks like, but they are not able to translate this into an explicit function [9]. This leads to the researcher having to spend extensive time on reward engineering, to create a reward function that enables the agent to learn the desired behavior.

One solution to the problem can be found in Human-in-the-loop (HITL) Reinforcement Learning.[10] Here somewhere in the training loop, human behavior or feedback is used to better align the agent’s behavior with the human’s expectations. An effective solution to that problem involves learning the policy implicitly through methodologies such as imitation learning and behavioral cloning [11, 12]. In these approaches, the agent learns by imitating the actions of an expert, allowing it to grasp the nuances of the task without explicitly defining a reward function. An alternative strategy is inverse reinforcement learning, by inferring the underlying reward structure from observed expert behavior, the system learns a reward model that should match more closely with the expectation [13]. Lastly, preference learning can be used to actively incorporate human feedback into the RL training loop [9]. This integration can occur directly, as demonstrated by methods like Deep Preference Optimization (DPO) [14], or indirectly through the creation of a reward model based on human feedback [15]. In most applications, the user ranks two or more outputs by their preferences and iteratively the model aligns with the expectations of the user

In many popular de novo drug design frameworks, the chemist must also express his preferences in the form of a parametric reward function, that describes the properties that chemists expect the generated molecules to have [16]. Chemists can struggle in defining well-specified reward functions. Organic and medicinal chemists are often not overly familiar with potential molecular descriptors that can be used to create a reward function. Additionally, they are not trained to think about molecules as a sum of individual properties. Rather they evaluate the quality of molecules more holistically by looking at the structural formula. This leads to a situation in which the reward functions produce molecules that are not aligned with the ideas of the chemists, and as a result, extensive manual cleaning and filtering of the generated molecules is necessary.

In generative chemistry, preference learning has been applied to mitigate the underspecification of reward functions. For instance, projects like MolSkill leverage human preferences to guide the generation of molecules [17]. A different study uses the liking or disliking of molecules to extract which property ranges are acceptable to chemists [18]. However, chemists often possess nuanced opinions about molecules, extending beyond the binary decisions of liking or disliking. They can provide valuable and specific feedback on properties and substructures, enabling a more nuanced understanding of the desired molecular characteristics. Collecting such specific feedback cannot only align the de novo design agent with human preferences, but in the long-term one can elucidate the implicit knowledge and experience of the chemist.

To capture this nuanced feedback from chemists, we have developed Metis, a user interface that facilitates the integration of specific and detailed human feedback into the RL process. Metis enables chemists to communicate their preferences, concerns, and insights, thereby enhancing the RL agent’s ability to generate molecules that align more closely with the desired properties and characteristics. Through Metis, we aim to provide an interface that enables practical Human-in-the-loop de novo drug design, ensuring a more effective and collaborative approach to molecular generation. To our knowledge Metis is the first GUI that allows the collection of such detailed feedback. MolSkill uses a Javascript-based UI in which two molecules are shown, the only input that can be given by the user is which of the molecules they prefer. In the work by Sundin and colleagues [18], a web application built with Streamlit was built called *MolWall*. Here multiple molecules are shown to the user, each of which they can rank on a Likert scale. Both of these GUIs do not allow feedback on specific properties or substructures.

With Metis, our objective is to provide a first-of-its-kind interface that enables practical Human-in-the-loop (HITL) de novo drug design. Additionally, it should serve as an initial step for research surrounding the methodology of HITL drug design, ultimately ensuring a more effective and interactive approach to molecular generation and closing the gap between the chemist’s expectation and the generative model.

## Application overview

**Fig. 1:**
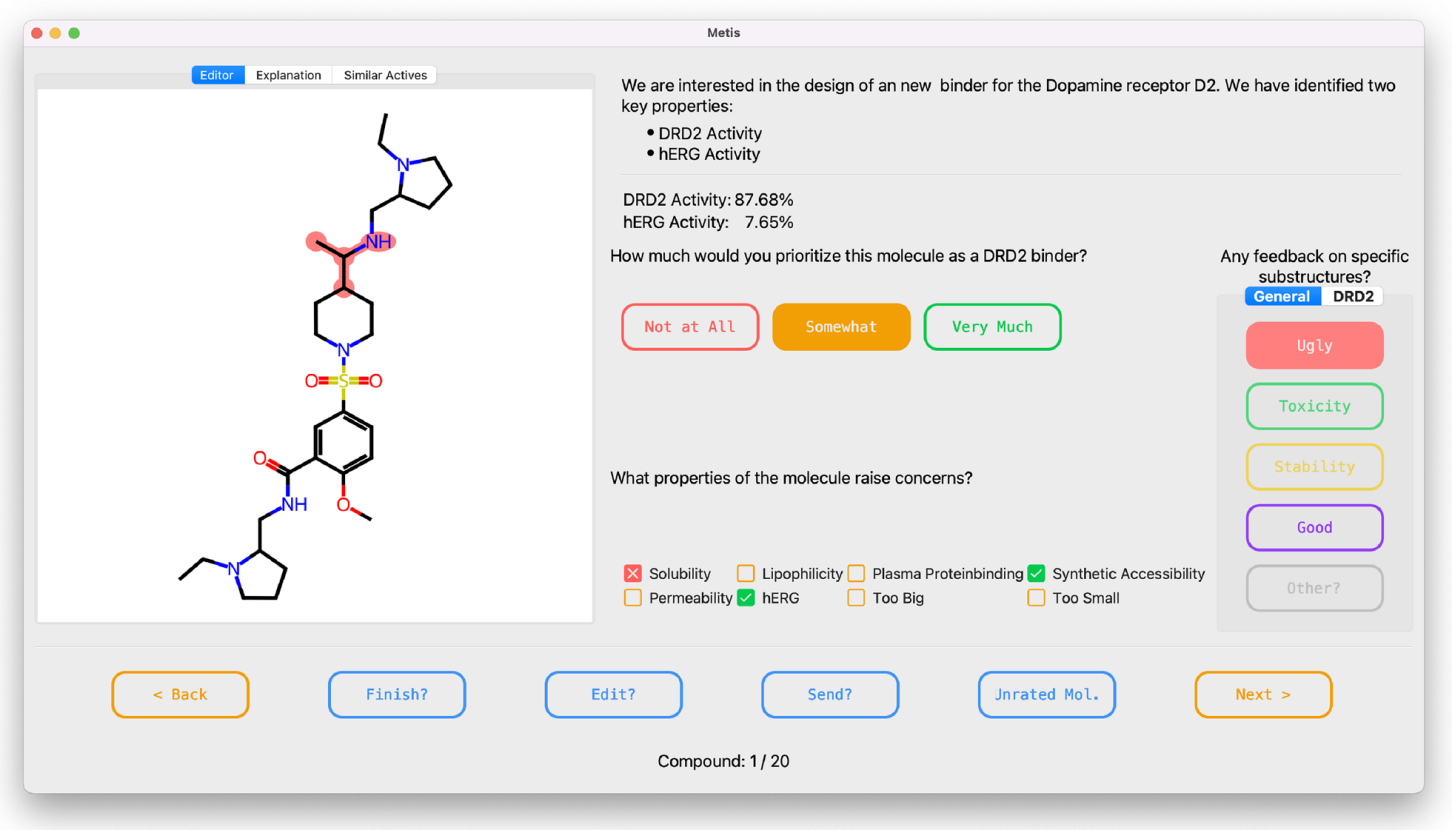
View of the Metis GUI

**Fig. 2:**
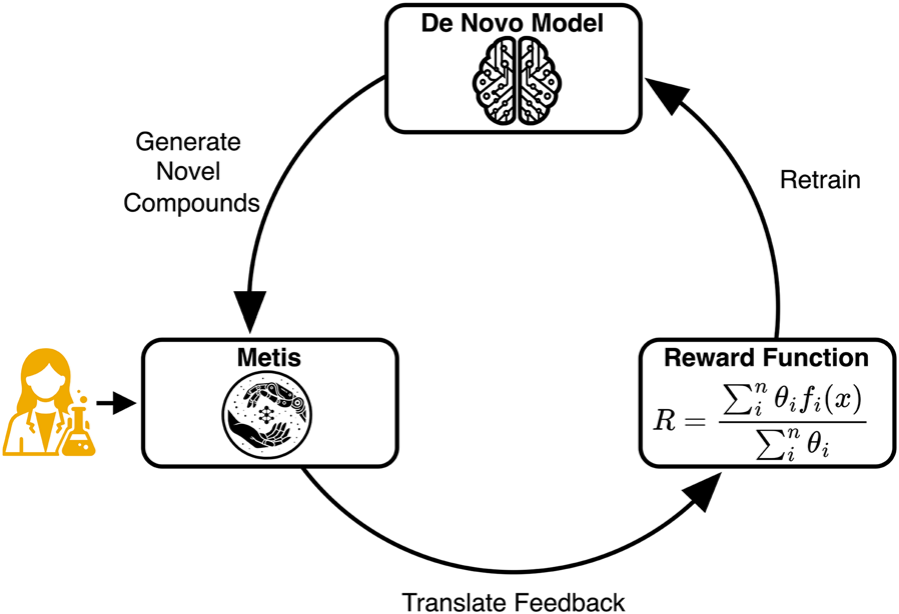
Overview of iteratively aligning the de novo model with the vision of the chemist using Metis

**Fig. 3:**
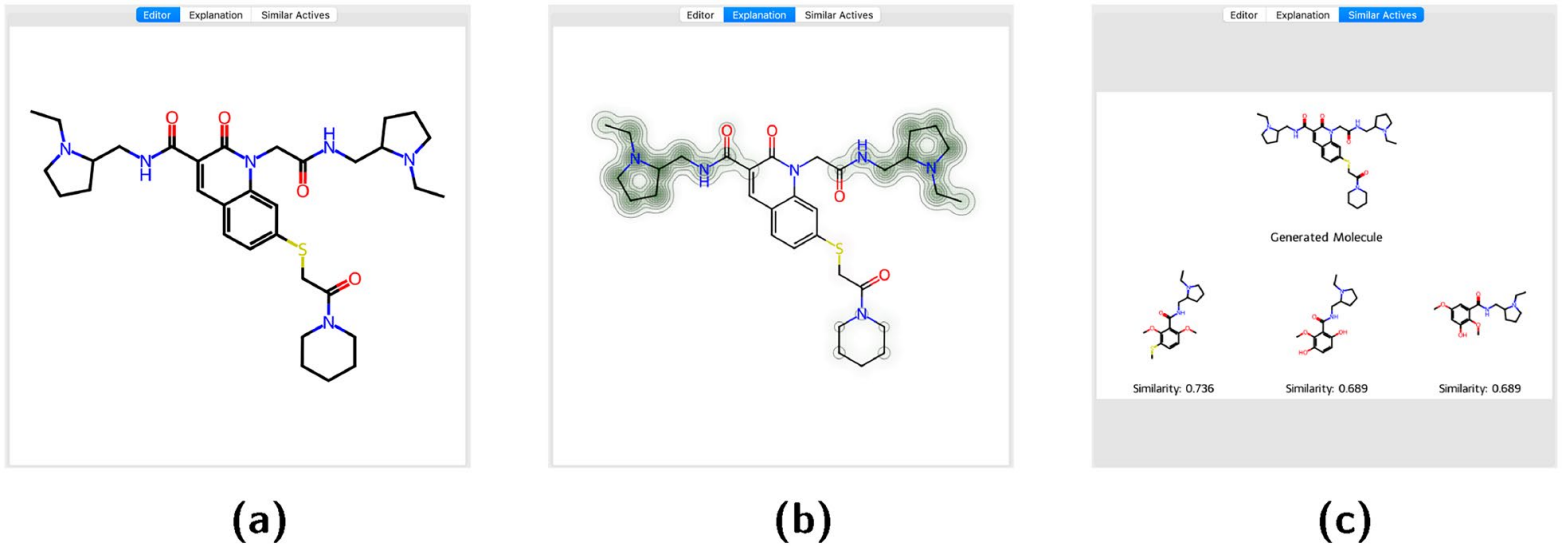
Showcase of the three windows the user can cycle through. **a** is the default window in which the user can select atoms to highlight. **b** the explanation window shows the per atom contribution to the prediction of an ML model and **c** is the window that shows the most similar active compounds from the training set

Metis is designed to allow (medicinal) chemists to provide feedback on small molecular structures. In particular, it is focused on collecting feedback on De novo-generated molecules. While chemists have general pre-disposition towards specific substructures, in practice the molecules are not evaluated in a vacuum. Rather, the chemists work in the context of a specific project. Typically, these projects involve targeting a specific protein for which an active molecule needs to be developed. Additional constraints such as solubility, selectivity, and toxicity may be specified. Given the dynamic nature of projects, a chemist’s preferences may vary significantly from one project to another. Hence, it is essential to collect and interpret feedback within the context of the project. To account for this Metis, does not only allow the Chemist to give feedback but can also provide project-relevant information to the chemists. However, Metis can not only be used to collect feedback but can also directly integrate this feedback into a *de novo* drug design run, which, if done in an iterative manner should align the generated molecules with the preferences of the user (Figs. 1, 2 and 3).

In the following the different components of Metis will be introduced in more detail.

### Feedback to de novo design

Metis can seamlessly re-integrate feedback back into a de novo design loop. The newly generated molecules using the feedback will then automatically be loaded into Metis and further feedback can be given. This loop allows the chemist to iteratively fine-tune their feedback as well as the molecular generator. Over multiple feedback rounds, the de novo design model should more closely align with the preferences of the chemist. Currently, Metis offers two methods for integrating user feedback into the de novo design model. One approach involves utilizing a *Reward Model*, a machine learning algorithm trained on the chemist’s feedback in the form of binary decision (like vs. dislike).. This model predicts whether a given molecule will be favored or disfavored by the chemist, thus contributing to the refinement of the de novo model. Presently, Metis supports all scikit-learn models utilizing an RDKit Morgan fingerprint.

An alternative method is to directly build a reward function from the feedback of the user. This reward function constitutes a sum of multiple equally weighted properties. At its core, it tries to minimize the presence of substructures flagged by the chemist as liabilities and tries to maximize the presence of favorable substructures. Additionally, it seeks to enhance similarity to liked molecules up to a specified threshold while minimizing similarity to disliked ones. In comparison to the reward model, the use of a reward function enables the integration of more fine-grained feedback that goes beyond liking versus disliking a molecule.

The extact reward function *g*(*x*) is defined as such:

$$\begin{aligned}g(x) = \frac{1}{6}\gamma ^+(x) - \gamma ^-(x) + \delta ^+(x) - \delta ^-(x) + 0.5\delta ^{\pm }(x) + \phi MS(x) \end{aligned}$$

where $\gamma ^+(x)$ and $\gamma ^-(x)$ calculate the number of liked/disliked substructures in molecule *x*. $\delta ^+(x)$, $\delta ^-(x)$, and $\delta ^{\pm }(x)$ calculate the Tanimoto similarity to liked, disliked and sort of liked molecules using the ECFP4. The similarity must be greater than 0.5 otherwise the resulting score will be 0. Finally, *MS*(*x*) returns the molecular size, the number of heavy atoms, $\phi$ is a double sigmoid function, that defines the acceptable range of the molecular size. The parameters are initialized to cover a wide range, but as feedback is given on the molecular cutoff values are iteratively updated in the direction of the feedback. The reward function is then added to the regular user-defined scoring function (*f*(*x*)) that is used in the current REINVENT run, thus the final scoring function *S*(*x*) amounts to:

$$\begin{aligned}S(x)=f(x) + g(x)\end{aligned}$$

This is a rather simple form to translate feedback from the human into a parametric function, but custom, more advanced approaches can also be integrated into Metis.

For now, only REINVENT [19] is supported. In order to make use of that feature, a working REINVENT Installation needs to be present on a remote machine, to which the users have access through an SSH key.

### Molecular display

The molecular display provides an image of the generated molecule, for which feedback should be collected. Users can click on atoms to highlight substructures within the molecule. Additional tabs offer users more detailed information about the molecule. The “Most Similar Active” tab allows users to see known active molecules most similar to the generated one, allowing chemists to judge whether the generated molecule is a sensible extension based on already known information. Additionally, the “Explainability” tab provides insights into why a scikit-learn [20] QSAR model suggests a generated molecule as potentially active, empowering chemists to make informed decisions based on the model’s reasoning. Currently, only the RDKits [21] native explainibility function developed by Riniker and Landrum [22] is available. But an extension to other methods should be easy to implement.

### Target product profile

The Target Product Profile window can be used to present information on the project at hand. Here are relevant properties and their relevancy can be explained. Descriptors that relate to these properties can also be shown for each molecule displayed.

### Global liabilities

The Global Liability window collects feedback on the overarching properties of a molecule. These global liabilities encompass characteristics not tied to specific substructures but rather arise from the molecule’s overall structure. For instance, the molecule’s size falls under this category. Additionally, it accommodates liabilities challenging to assign to specific atoms, such as synthetic accessibility, which can be easier to evaluate globally. Chemists can assess each property showcased and determine if the molecule aligns with their conceptualization of it or not. Crucially, within this window, chemists can provide feedback on their overall preference, whether they like or how much they like this molecule.Currently, most of the feedback on specific liabilities can not be integrated into the de novo design loop, as there is no straightforward way to computationally assess this liability. For example, permeability is difficult to predict. For, easier properties, like the size of the molecule, the initial range of “allowed sizes” of molecules is very wide. As feedback regarding size is given, the range is shifted. Even if the feedback is not usable in a de novo design run, the recording of it is very crucial, as given enough data one can create rule-based or machine learning-based model to predict these liabilities

### Local Liabilities

Local Liabilities refer to liabilities of molecules that can be directly mapped to specific local substructures. Users have the flexibility to toggle between different liabilities they wish to highlight within the molecule. By selecting atoms in the molecular display, these substructures can be associated with the corresponding liability, each distinguished by a unique color. Additionally, users can create new labels, not predefined, by specifying their concerns for a particular substructure in a text field. Not technically a liability, but by default Metis also provides the chemist the option to highlight substructures that they like. The chemist can also make a distinction of whether the feedback he provides is feedback that is relevant only to the current project, or whether the feedback is generally valid across many projects. The highlighted substructures are stored and saved by recording the atom indices of highlighted atoms, additionally, the substructures are directly mapped to a SMARTS pattern that is also saved. Next to the atom and bond type, the SMARTS pattern also recorded ring membership and the number of attached hydrogens for each atom. To ensure that adequate information is recorded, the SMARTS pattern is expanded to also include all atoms that are directly connected to the highlighted substructure. This way the feedback of the chemist can more adequately be saved. The significance of this approach becomes evident when considering examples such as distinguishing between amides and ketones. If a chemist is dissatisfied with a ketone, they are likely to flag the atom and the double-bonded carbon but may overlook the two additional carbons. Further down the line, this can lead to desirable amides being flagged as liabilities. By recording the expanded environment of a highlighted substructure, such oversight is mitigated, ensuring that chemists’ feedback is accurately represented.

Like with the global liabilities, we can currently not distinguish in an de novo run between different kinds of substructures “critiques”. Liked substructures will increase the rewards and disliked substructures will decrease the reward of a given model, independently of why the substructure was disliked.

### Additional features

In the navigation bar at the bottom of the GUI, multiple additional helpful buttons are provided. Most importantly, the “Next” and “Back” buttons allow the users to switch between molecules that are supposed to be evaluated. The “Edit” button will open a molecular editor in a separate window. The editor can be used by the user to suggest an alternative molecule to the one that is currently to be evaluated. The editor will open with the current molecule already loaded. The molecular editor that is used is the rdEditor[23] Changes made to the molecule in the editor will then be stored separately in the backend. The “History” button will also open a separate window, in which the chemist can scroll through the already evaluated molecules. Lastly, the “Send” button, will start a new de novo run on a remote machine using the feedback provided by the Chemist. A more detailed description is found in the following section.

## Customizability

Metis is designed to be customizable through a yaml file, in which the users can specify which information to show to the chemist, and what kind of feedback can be given by the chemist. The exact liabilities can be changed, but also complete GUI elements can be removed if needed. A complete list of all settings and their use, together with some examples are provided with the GitHub Repository. The examples are designed around a fictitious drug design project around designing a MAPK10 (JNK3) kinase inhibitor. For this, initial molecules were generated with REINVENT. The generated molecules as well as the models are provided with examples. The examples provided three different setup files to cover different use cases and complexities in setting up the GUI.

1. **UI Only Example** In this example, Metis is only used to collect feedback for generated structures. No models are re-trained and no de novo run can be started.
2. **Reward Model Example** In addition to setting up the GUI, here the feedback is used to directly train the reward model. However, still, no de novo run can automatically be started. This setup can be useful, in scenarios where one is only interested in building a reward model or the reward model shall be used in a different de novo environment.
3. **De Novo Loop Example** This example showcases all the functionalities of Metis. The user feedback is collected, a reward model is trained and subsequently used to start a de novo run using REINVENT on a remote machine. The newly generated structures are then copied and loaded into Metis. While the other two examples can be started immediately. This example requires REINVENT installation on a remote machine and some files need to be transferred.

As Metis is written in Python changes to modules not “exposed” through the yaml settings file, can also be changed by adding additional classes that follow the design of already implemented classes. Examples of such are classes that take care of the sampling of molecules, or how the reward models are trained.

For the iterative re-training of the de novo models, one could in theory use any de novo model. However, Metis does soft-lock users to use REINVENT. The limiting factor is that no unified standard for de novo design tools has been proposed or adopted. Thus, most models require different setups with different configuration files, which then return their results in different file formats. This makes it difficult to ensure operability between different models. While it is possible to use an alternative to REINVENT, it would require significant modifications by the user to the existing code of Metis.

## Implementation

Metis is written in Python and relies at its core on three libraries. PySide2 [24] is a Python implementation of the Qt Framework and is used to create the Graphical User Interface (GUI) a user can interact with. The molecular drawing, highlighting and editing capabilities are provided by rdEditor [23]. Additionally, RDKit is used to manipulate, and save molecular structures and information. Further core libraries in use are pandas, numpy, and scikit-learn.

**Fig. 4:**
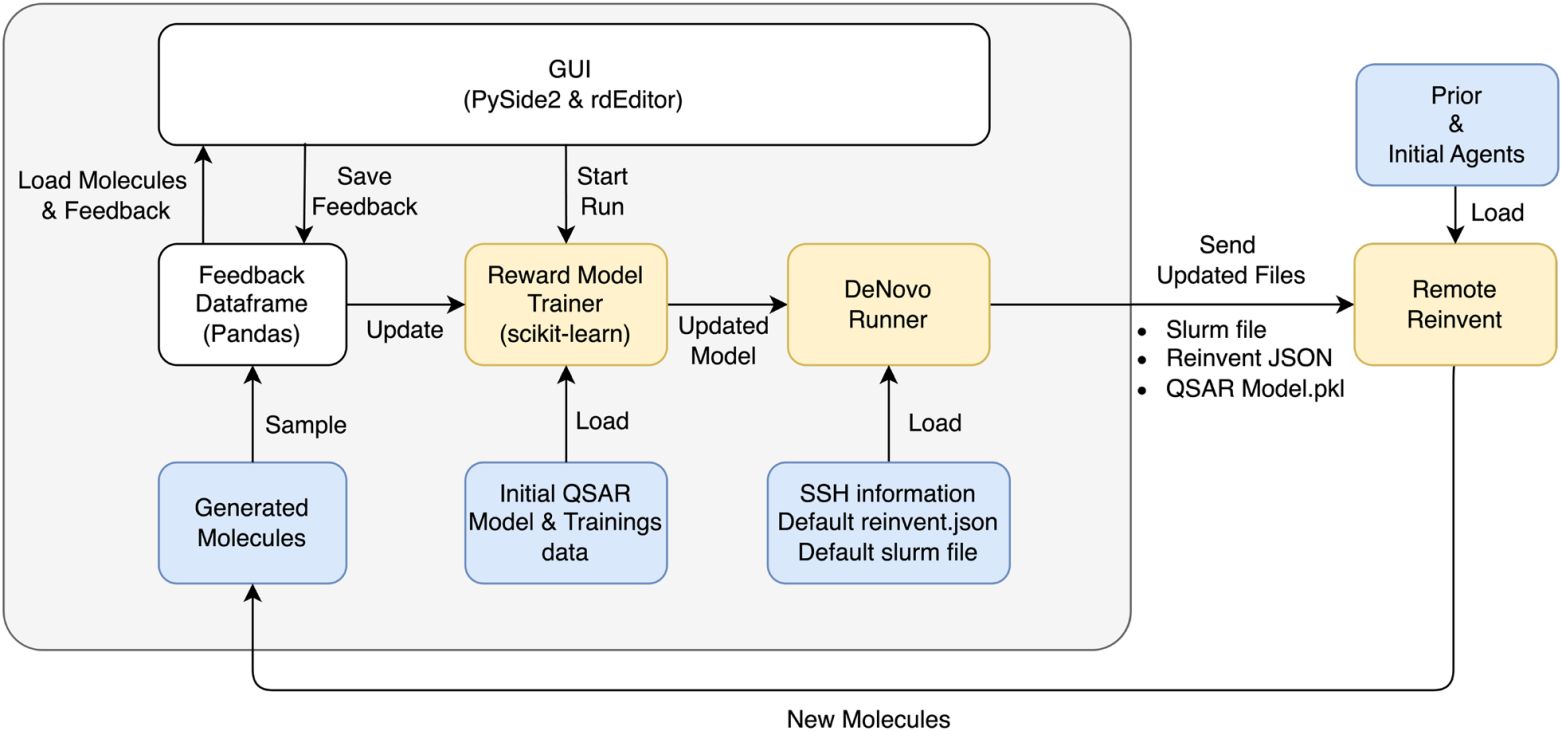
Schematic overview of Metis. Yellow squares indicate modules that are only optional and only needed if Reward Models should be trained and/or de novo model run should be started

Figure 4 gives an overview of how the different parts of Metis come together. The interface the user can interact with is created using the aforementioned PySide2 and rdEditor. Molecules and their associated feedback reside within a custom extension of a pandas data frame. This specialized data frame efficiently stores diverse forms of feedback and translates between atom indices and their corresponding SMARTS structure representations. The molecules presented to the user are sampled from an initial set of molecules stored in a separate file.

The “Reward Model Trainer” class handles both the training of the reward model and the creation of the reward function. If a QSAR Model exists that needs fine-tuning with user feedback, the trainer loads the QSAR model along with its original training data and merges it with the obtained feedback. Subsequently, the model undergoes re-training using the combined dataset. In the absence of an initial model, training commences from scratch.

For a de novo run, the process involves initializing a “De Novo Runner” class instance on a separate core. This runner then generates input files for REINVENT and transfers them, along with the updated model in a “pickle” format, to the remote location via SSH. A remote run is then initiated using SLURM. The “De Novo Runner” remains in a waiting state until the REINVENT run concludes, after which it retrieves the current state of the Agent and the newly generated molecules back to the local machine. From here, new molecules are selected to be evaluated by the user.

In each iteration, the molecules, their feedback, the reward models, and the de novo model are saved.

### Installation

Metis is an open-source software that can be downloaded from https://github.com/JanoschMenke/metis. After setting up the environment either manually or through the use of the pyproject.toml file, the software can be used. A more detailed description of the setup and the settings is provided on the GitHub repository, together with examples that should let the user get started directly.

### License

Metis is published under the permissive MIT license.

### Limitations

The sole reliance on Python, Pyside2, and RDKit makes Metis very adaptable for all researchers in cheminformatics and its adjacent fields. Most researchers code in Python and are able to make their desired changes. However, this choice for PySide/Python, also makes Metis not currently hostable on the web. This can be attributed to the fact that PySide2 at its core uses C++ and currently does not have WebAssembly support. The second limitation, previously mentioned, is that by default only REINVENT as a de novo model is supported. While it is not difficult for users to adapt the code to their model of choice, many small changes need to be made, as Metis is written with REINVENTs file formatting in mind. Lastly, as mentioned previously much of the very detailed feedback that is collected is currently used in the de novo design loop, as the property for which we collect feedback is not a property that can easily be measured or predicted. However, collecting and saving this feedback is still valuable to build models later around such properties.

## Outlook

There are still some features not yet integrated that would enhance the usability of Metis. We aim to upgrade the support to the recently released REINVENT 4 [25] and in the long-term provide some interface layer, which makes it easier to also include non-REINVENT de novo design tools. We also plan to expand support to scikit-mol [26] models, which can be used to train the reward model. This provides more customizability to the user with regard to the selection of input features and model choice. We further hope to extend support to the recently released REINVENT 4 and while we do not think any de novo design tool can be supported without manual editing of the code we want to make the interface easier to manipulate so other de novo tools are easier to connect to Metis.

## Conclusion

Here we introduce Metis, a Graphical User Interface, that enables researchers to collect feedback on generated molecules that go beyond simple like or dislike. Chemists can assign substructures specific liabilities, flag concerning properties, and can suggest alternative molecules to the generated ones. Metis also serves as a platform to provide chemists with sufficient information on the task to make informed decisions on the generated compounds. As the feedback can be directly integrated within existing de novo Design loops, the GUI has its practical application and can help end-users to fine-tune and align the generative model with their ideas and preferences. To our knowledge, no other application exists that provides such functionality and Metis can serve as a starting point for the community to develop and test ideas on how elaborate chemical knowledge and the feedback it gives rise to, can adequately be modeled and integrated into existing deep learning models.

## Data Availability

All code, and data used required for testing the GUI, are free and publicly available from https://github.com/JanoschMenke/metis.
